# Molecular Research on Platelet Activity in Health and Disease 2.0

**DOI:** 10.3390/ijms22094968

**Published:** 2021-05-07

**Authors:** Maria Valeria Catani, Isabella Savini, Valentina Tullio, Valeria Gasperi

**Affiliations:** Department of Experimental Medicine, Tor Vergata University of Rome, 00133 Rome, Italy; savini@uniroma2.it (I.S.); valentinatullio.nu@gmail.com (V.T.)

## 1. Original Articles

Hsia and collaborators [1] describe the ability of auraptene, the most abundant coumarin derivative from plants, in reducing platelet activity, via NF-κB-dependent reduction in phospholipase C γ2(PLCγ2)-protein kinase C (PKC) activity. Murata’s group reports that rocuronium, a steroid-based drug widely used for anesthesia and intensive care, antagonizes, via its morpholin ring, platelet aggregation, cAMP production and P-selectin surface exposure, all events triggered by P2Y12 receptor stimulation [2]. 

Further insights into molecular mechanisms underlying platelet biogenesis are provided by Van Dijk and collaborators, who address the role of p21-activated kinase 1 (PAK1) in microtubule acetylation and cytoskeleton integrity. Through a validated cell model of proplatelet formation, elongation and platelet release, the authors demonstrate that PAK1 directly inhibits the α-tubulin acetyltransferase (MEC-17), thereby guaranteeing correct microtubule acetylation levels and cytoskeleton integrity [3].

Pielsticker and collaborators investigate the role of platelets in inflammatory processes, by focusing on interaction between platelets and the fibrinolytic system. They show that the fibrinolytic enzyme plasmin dose-dependently triggers P-selectin exposure on membrane surface and fibrinogen binding to human platelets. They also identify several factors regulating the platelet-activating potential of plasmin; among them, deserving of special mention is thrombospondin-1 that, depending on its concentration, can act as cofactor or inhibitor of plasmin-induced platelet activation [4].

Kraemer and colleagues [5] explore platelet proteome changes in response to laminin, collagen or fibrinogen, in order to explain stimulus-dependent differences in hemostatic and migratory responses of activated platelets. They report that phenotype and cytoskeletal patterns in activated platelets are distinct on the base of extracellular matrix components. Interestingly, mass spectroscopy analysis demonstrates that laminin-adherent platelets specifically upregulate proteins that are essential for migration, such as coronin or kindlin-3, filamin A, vinculin, talin and gelsolin. 

Platelet proteome changes are also associated with different pathological conditions, including diabetes. Through mass spectrometry analysis, Kyselova and collaborators demonstrate that platelets from diabetic patients show a reduction in cyclin Y (CCNY), a well-known regulator of integrin-mediated outside-in signaling associated with thrombin stimulation. Consequently, although CCNY/mice have fewer circulating platelets, nonetheless their platelets respond to thrombin and to a thromboxane A2 analog in a superimposable manner to platelet from wild-type mice. In addition, platelets from KO mice show increased adhesion to fibronectin and collagen, as well as attenuation of clot diffusion and retraction. Collectively, these phenomena suggest an alteration in integrin signaling “out” [6]. 

Particularly intriguing is the role of platelets in multiple sclerosis (MS), an autoimmune and degenerative disease of the central nervous system. Epidemiological studies have proved that comorbidities, such as ischemic stroke, atherosclerosis, thrombosis, and myocardial infarction, are associated with increased mortality in MS patients, especially in the progressive stage of MS (SP-MS). As exposed by Dziedzic’s study, SP-MS patients display increased formation of platelet aggregates, platelet-derived microparticles and platelet-leukocyte aggregates, as well as greater surface P-selectin expression, with respect to control subjects. Such hyperactivity is related to increased pro-thrombotic platelet activity, as shown by higher protease-activated receptor (PAR) expression. Finally, the finding that, in MS megakaryocytes and platelets, the expression of mRNA transcripts for some atherosclerosis biomarkers (such as APOA1 and A2M) correlates with PAR expression emphasizes that alterations of cholesterol pathway biomarkers are linked with MS progression towards the neurodegenerative phase [7]. Further insights into the role of PAR1 in platelets derive from Rabani and colleagues, who show that lipid rafts contribute to PAR1 signaling in platelets, despite a weak localization of the receptor in these cholesterol-rich domains [8].

Platelets are also involved in various preclinical and clinical scenarios of liver regeneration, after injury and resection. Platelets, indeed, can interact with liver sinusoidal endothelial cells and release serotonin, ADP, and other factors that directly stimulate hepatocyte proliferation. Lehwald and colleagues demonstrate that platelets increase hepatic homing of CD133^+^ bone marrow stem cells (BMSC) to injured liver, through a mechanism dependent on interaction between P-selectin and its ligand P-Selectin-Glyco-Ligand-1 (PSGL-1). In light of these data, platelets, therefore, may be used to maximize the efficacy of BMSC treatment in liver regeneration, as well as in other hepatic diseases, such as hepatitis and liver cirrhosis [9]. Platelet factors have been reported to promote proliferation not only in hepatocytes, but also in other cell types, including chondroprogenitors and chondrocytes. Nguyen and colleagues investigate, indeed, the role of some platelet-releasing molecules in the physiological response of bone cells to injury. They show that, in conjunction with bone damage, resident precursor cells reactivate proliferation and expansion. Furthermore, platelets activate STAT3, thus stimulating endothelial cells involved in angiogenesis and osteogenesis processes. These results corroborate the finding that, in regenerative medicine, platelets activate quiescent osteoblasts, while stimulating angiogenesis [10].

## 2. Reviews

Chatterjee and collaborators exhaustively provide an update of research on GPVI activation, GPIb-vWF interactions, regulation of integrin signalling, chemokine receptors, and channel homeostasis of PANX1 and NMDAR, highlighting that these receptors, involved in platelet activation, may represent new molecular targets for anti-thrombotic and anti-thrombo-inflammatory therapies [11]. Braune and colleagues discuss the important role of prostanoids [including prostaglandin-D2 (PGD2), prostaglandin-E1, -E2, and E3 (PGE1, PGE2, PGE3), prostaglandin F2 (PGF2), prostacyclin (PGI2), and thromboxane-A2 (TXA2)] in hemostasis by regulating blood platelets, through both inhibitory and activating mechanisms, depending on the type of prostanoid [12]. Other factors affecting platelet activity are Toll-like receptors (TLRs), which are widely expressed in several cell types, including platelets. Hally and collaborators provide an update on the role of platelets and TLRs in inflammation and infection. They clearly illustrate that platelets behave as both thrombotic and immune cells, acting as blood sentinels to detect and counter endogenous and exogenous risks [13]. Furthermore, platelets can communicate with other cells, through the production and release of chemical mediators. Dovizio and collaborators, in their narrative review, extensively examine the role of platelets in communicating with cancer cells, a phenomenon known as tumor-educated platelets. Platelets can uptake proteins and transcripts from circulation, thereby showing specific proteomic and transcriptomic expression patterns. Therefore, the transcriptomic and proteomic platelet profiles may be useful for early diagnosis, as well as for monitoring specific therapies. Thanks to their ability to interact with cancer cells, platelets have been used in drug delivery to reduce toxicity and increase drug efficacy. For these reasons, platelets could be combined with traditional chemotherapy to improve cancer diagnosis and treatment [14].

An important factor that can improve the prognosis in cancer patients is the platelet count. Hufnagel and co-workers provide current evidence for associations between thrombocytosis and ovarian cancer. They analyze the clinical importance of platelet count thresholds and discuss several clinical studies, as well as in vitro and in vivo mechanisms that may be involved in this association. These data highlight that regular monitoring of platelet counts, during and after therapy, may represent a possible way for assessing response to therapy, disease progression, or relapse [15].

Platelets also play an important role in depression. Amadio and colleagues analyze some molecules, including adipokines, catecholamines, lipids, chemokines, and reactive oxygen species, potentially associated with platelet activation and aggregation in depressive disorders. Neurons and platelets are different, but they have similar characteristics: platelets can respond to changes in serotonin levels. The authors show that platelets are activated in response to serotonin and in depressed patients. These data demonstrate how platelets may be involved in increased risk of cardiovascular disease in depressed individuals [16].

Another important aspect in platelet research concerns clinical applications of platelet concentrates for regenerative medicine. This is the subject of the systematic review by Gentile and colleagues, who analyze studies on the concentration of platelet-rich plasma (PRP) in vitro and the in vivo effect of PRP on tissue regeneration. They show that a PRP concentration of 1.0 × 10^6^ plt/µL is optimal for abundant capillary nutrition and an optimal PRP/media ratio to prevent cell hunger is PRP 10% (vol/vol). From these considerations, it emerges that the near future will probably be based exclusively on allogeneic and/or autologous regenerative therapies [17].

Antiplatelet drugs have so far been widely administered to patients with cardiovascular disease. As our understanding of the biological role of platelets continues, new drug classes will be produced, not only for treatment of cardiovascular disease, but also for cancer and other inflammatory disorders. All studies reported in this Special Issue reveal new aspects of platelet biology that could be of great use in the development of new therapeutic strategies. We hope that they will be useful for new insights into the management of pathological conditions dependent on platelet dysfunction.
